# The relationship between ankle quasi-stiffness and postural stability in older adults during obstacle negotiations using the dominant and non-dominant sides

**DOI:** 10.3389/fbioe.2026.1715276

**Published:** 2026-06-04

**Authors:** Shikun Wang, Chen Yang, Quan Zhou, Yan Chen, Chuanxia Zhang

**Affiliations:** 1 School of Sports and Health, Shandong Sport University, Jinan, Shandong, China; 2 College of Physical Education and Sport Science, Qufu Normal University, Qufu, Shandong, China; 3 Department of General Education, Shandong Xiehe University, Jinan, Shandong, China

**Keywords:** ankle stiffness, limb asymmetry, margin of stability, obstacle negotiation, older adult

## Abstract

**Background:**

In this study, we aimed to analyze the differences in ankle quasi-stiffness between the dominant and non-dominant sides during obstacle negotiations in older adults and examine their relationship with postural stability.

**Methods:**

A total of 16 older adult participants performed 10 obstacle-crossing trials with each limb. The margin of stability (MoS), ankle quasi-stiffness, and integrated electromyography of the tibialis anterior, soleus, and gastrocnemius lateralis in the stance limb were analyzed.

**Results:**

Negative correlation was found between frontal-plane stiffness (*p* = 0.007, r = −0.371) and sagittal-plane stiffness (*p* = 0.027, r = −0.306) with the MoS under high fall-risk conditions during dominant-side negotiations. A similar negative correlation was found under low fall-risk conditions, with frontal-plane stiffness (*p* = 0.041, r = −0.473) and sagittal-plane stiffness (*p* = 0.001, r = −0.676) correlating with the MoS. Additionally, a positive correlation was observed between frontal-plane stiffness (*p* = 0.005, *r* = 0.361) and the MoS under high fall-risk conditions during non-dominant-side negotiations.

**Conclusion:**

During obstacle negotiation, when using the dominant side, ankle quasi-stiffness was lower to enhance postural stability, whereas when using the non-dominant side, ankle quasi-stiffness was greater for faster obstacle negotiation. These findings highlight distinct limb-dominance-based strategies when dealing with complex dynamic tasks. Future training methods and assistive device designs should be specifically targeted to the distinct functional roles of each limb.

## 1 Introduction

Falls are a major health risk for adults aged over 65 years worldwide. Notably, approximately one-third of the fall incidents are caused by challenges encountered during obstacle negotiations (Blake et al., 1988; Campbell et al., 1990). Previous studies have identified several variables correlated with postural stability during obstacle negotiations, including toe clearance, cognitive load, lower-limb muscular strength, joint coordination, and other factors (Dubbeldam et al., 2023/02; Kang and Lipsitz, 2010; Yamagata et al., 2021; Cherif et al., 2020). Ankle quasi-stiffness may correlate with postural control. Quasi-stiffness is defined as the slope between the joint angle and the normalized joint internal net moment (MacLean and Ferris, 2022). It is influenced by both the active and passive properties of the muscles and tendons that span the joint; activation of these muscles or stretching of these tendons typically increases quasi-stiffness.

Researchers have performed studies on the relationship between ankle quasi-stiffness and postural stability in activities of daily living. Increased quasi-stiffness is associated with decreased postural stability during quiet stance (Vette et al., 2017). During dynamic tasks, quasi-stiffness also influences postural stability. Older adults tend to decrease ankle quasi-stiffness during stair descent to enhance flexibility and improve balance adjustment (Ma et al., 2023; Lark et al., 2003). Moreover, older adults’ quasi-stiffness decreases with reduced gait speed due to decreased ankle plantarflexion moment and increased dorsiflexion angle (Collins et al., 2018). Because obstacle negotiation requires advance processing of visual information, gait speed decreases (Maidan et al., 2018; Galna et al., 2009), potentially causing decreased quasi-stiffness. However, the relationship between ankle quasi-stiffness and postural stability in older adults during obstacle negotiation remains unquantified.

Obstacle negotiation is a common daily activity for people (Kolk et al., 2014; Shin et al., 2015), who prefer to use the dominant side (DS) for obstacle negotiation, as it possesses the characteristics of shorter reaction time and higher accuracy when faced with obstacles (Bhise and Patil, 2016). They also sometimes use the non-dominant side (NDS) to cross obstacles. Previous studies have reported that older adults show impaired performance when they are required to negotiate obstacles using their non-dominant side (Orcioli-Silva et al., 2020), and older adults with greater asymmetry during obstacle negotiation using both sides tend to have a higher fall risk (Di Fabio et al., 2004). This impaired performance may be associated with the distinct functional roles of the bilateral limbs during locomotion. Understanding the relationship between ankle quasi-stiffness and postural stability during obstacle negotiation would provide valuable insights for the design and development of methods to prevent falls, such as ankle–foot orthoses (AFOs), specifically to determine whether assistive devices should provide rigid support or facilitate adaptive stiffness modulation. For example, some researchers have developed the AFO for adjusting joint quasi-stiffness (Lecomte et al., 2021) and highlighted the correlation between quasi-stiffness during walking (Mahmood et al., 2022).

Therefore, the purpose of this study was to analyze the relationship between ankle quasi-stiffness and postural stability when older adults used their DS and NDS during obstacle negotiation. We calculated the margin of stability (MoS) as an indicator of postural stability (Curtze et al., 2024a), ankle quasi-stiffness, and integrated electromyography (EMG) of the tibialis anterior (TA), soleus (SOL), and gastrocnemius lateralis (GL) during obstacle negotiations in older adults. We hypothesized that (1) ankle quasi-stiffness would be negatively correlated with postural stability under DS and NDS conditions; (2) because of the distinct functional roles of the lower limbs, the NDS would exhibit greater ankle quasi-stiffness and poorer stability than the DS, reflecting a difference in strategy during obstacle negotiation with DS and NDS.

## 2 Methods

### 2.1 Participants

A total of 16 eligible older male adults (age: 67.64 ± 2.08 years, height: 168.96 ± 6.05 cm, and mass: 63.63 ± 9.64 kg) were recruited to participate in the study from Shandong Sport University and the surrounding community. The inclusion criteria were as follows: (1) over 65 years old; (2) right leg being the dominant side, which was confirmed by the Waterloo Footedness Questionnaire-Revised (WFQ-R) (Elias et al., 1998); (3) Mini‐Mental State Examination (MMSE) score ≥24 (Jia et al., 2021); and (4) able to live independently and perform the activities of daily living. The exclusion criteria were as follows: (1) a history of stroke or myocardial infarction; (2) alcohol consumption within 6 h prior to participation; (3) a history of falls in the past year; (4) visual acuity (or corrected) less than 0.5; and (5) the use of any medication affecting physical balance and the nervous system, such as sedatives and tranquilizers, in the last 6 months. All the participants provided written informed consent after receiving the detailed explanation of the study, which was approved by the Sports Science Ethics Committee of Shandong Sports University (grant number: 2022025). All experimental methods were performed in accordance with the latest guidelines and regulations of the Declaration of Helsinki.

### 2.2 Experiment protocol

**FIGURE 1:**
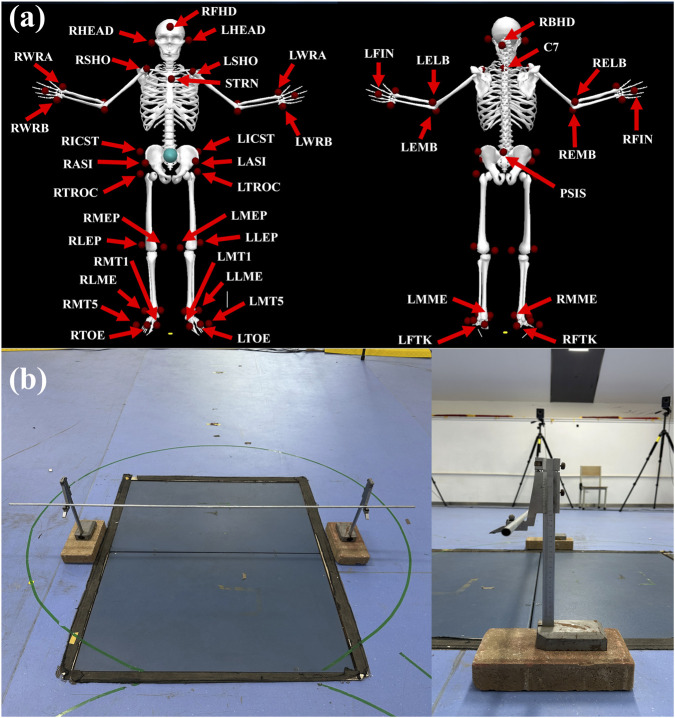
Marker placement and experimental setup. Experimental setup and marker placement. **(a)** This panel illustrates the location of 41 reflective markers affixed to the body for motion capture. **(b)** Obstacle and force platforms. The obstacle is constructed using an aluminum bar integrated with two adjustable supports, allowing for height adjustment. Two AMTI force plates are mounted flush with the floor and positioned beneath the obstacle.

The leg length of each participant was defined as the distance between the greater trochanter and the lateral malleolus. Then, 41 reflective markers were placed on the participant’s bony landmarks, as shown in Figure 1a. According to the SENIAM recommendations (Hermens et al., 2000/10), six surface EMG sensors (Ultium, Noraxon, United States) were attached to the bilateral TA, SOL, and GL to collect EMG data (sampling rate: 2,000 Hz). The motion-capture system with 12 infrared cameras (Vicon Vantage, Oxford, United Kingdom) was set up around the test area to collect kinematic data (sampling rate: 100 Hz). Two force platforms (BP600900, AMTI, United States) were mounted on the floor at the center of the test area to collect the center of pressure (COP) data (sampling rate: 1,000 Hz) (see Figure 1b).

The obstacle consisted of two upright stands with an unfixed crossbar and was placed in the middle of the force platforms (see Figure 1b). The obstacle’s length was 60 cm and the width was 3 cm, and the height was set to 20% of the participant’s leg length (Chardon et al., 2022). The starting position was located at a distance of 15 m from the obstacle. The participants used their DS and NDS, respectively, to negotiate the obstacle 10 times with sequential randomization. If the participant’s foot contacted the obstacle, the trial was discarded. The motion-capture system, force platforms, and EMG system were synchronized via cable. The tests where the dominant leg crossed the obstacle and the non-dominant leg supported the movement were termed DS negotiations; conversely, the tests with the non-dominant leg crossing and the dominant leg supporting were termed NDS negotiations in the following sections.

### 2.3 Data processing

**FIGURE 2:**
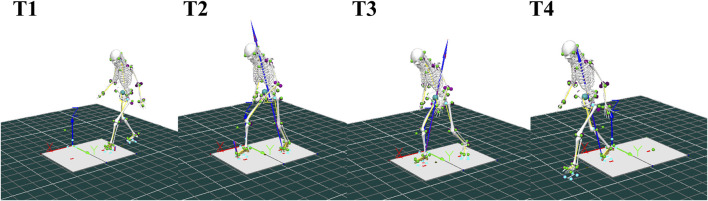
Phases and key events of obstacle negotiations. T1: trailing limb contacts with the force plate (Z-axis force >10 N); T2: leading limb contacts with the force plate (Z-axis force >10 N); T3: trailing limb is toe-off (Z-axis force <10 N); T4: trailing limb contacts the ground again.

Visual3D (V6 Professional, HAS-motion, Canada) software was used to build the rigid-body model and identify key events in the motion file, with labels placed at specific locations. T1 represents the trailing limb heel contacting the ground (Z-axis ground reaction force exceeded 10 N), T2 represents the leading-limb heel contacting the ground (Z-axis ground reaction force exceeded 10 N), T3 represents the trailing-limb toe leaving the ground, and T4 represents the trailing-limb heel contacting the ground again. T1–T2 represents the negotiation phase, which is the focus of this study (see Figure 2).

Marker trajectories were filtered using the fourth-order recursive low-pass Butterworth filter (cut-off frequency: 10 Hz) (Hak et al., 2019). Joint angles were calculated from the relative rotations between two adjacent segment coordinate systems, and joint moments were estimated using an inverse dynamics approach (Huang et al., 2024). Both the joint angles and moments were expressed using the Cardan sequence in Visual3D, which is a standard method for representing joint kinematics recommended by the International Society of Biomechanics (see Table 1). The mean speed of the center of mass (COM) in the anterior/posterior (AP) and medial/lateral (ML) directions was calculated as the COM velocity at the negotiation phase. Because the positive ML direction was defined as pointing to the right side of the stepping direction, the direction of the COM motion differed between obstacle negotiation performed with the DS and that with the NDS. Specifically, the COM motion occurred in the positive ML direction when using the DS and in the negative ML direction when using the NDS. Consequently, the COM velocity also showed opposite signs; therefore, absolute values were used for comparison and statistical analysis.

**TABLE 1:** Instructions of the cardan sequence in this study.

Joint	Segment (distal)	Segment (proximal)	Sequence X	Sequence Y	Sequence Z
Ankle	Foot	Shank	PF [-]/DF [+]	EV [+]/INV [-]	ER [+]/IR [-]
Knee	Shank	Thigh	EXT [+]/FLE [-]	VAL [+]/VAR [-]	ER [+]/IR [-]
Hip	Thigh	Pelvis	EXT [-]/FLE [+]	ABD [+]/ADD [-]	ER [+]/IR [-]

Abbreviation: PF, plantar flexion; DF, dorsiflexion; EXT, extension; FLE, flexion; EV, eversion; INV, inversion; VAL, valgus; VAR, varus; ABD, abduction; ADD, adduction; ER, external rotation; IR, internal rotation.

The mean value of MoS for the stance limb during T1–T2 was calculated according to the following equations from previous studies (Curtze et al., 2024a; Curtze et al., 2024b). In Equation 1, g represents gravity, which is 9.81 m/s^2^; l is the pendulum length, which is estimated as 1.34 times the distance between the lateral ankle and the greater trochanter markers; and is the natural frequency of the inverted pendulum model, see Equation 1:

$$\omega_{0}=\sqrt{g/\left(l*1.34\right)}.$$ (1)

In Equation 2, $D_{com}$ represents the coordinates of the COM, XcoM is the position of the COM accounting for velocity, and $V_{com}$ is the COM velocity, see Equation 2:

$$XcoM=D_{com}+\frac{V_{com}}{\omega_{0}}.$$ (2)

In Equation 3, $B_{\max}$ is the maximum value of the support margin, representing the coordinate of the COP from the force platform contacted by the stance limb, see Equation 3:

$$MoS=B_{\max}-XcoM.$$ (3)

In each condition (DS and NDS), trials with MoS values exceeding the upper quartile of all trials within that condition were classified as good postural stability, while the remaining trials were classified as poor postural stability (Sadeqi et al., 2023; Ueno et al., 2021). The rationale for this approach is twofold. First, due to the lack of established clinical criteria defining the optimal versus compromised MoS values during obstacle crossing, we relied on a data-driven method rather than arbitrary or subjective thresholds. Second, older adults typically show high motor variability; consequently, high-risk and low-risk trials often coexist within the same individual’s performance. A key advantage of this trial-based method is its ability to discriminate between these divergent states. This separation allowed us to identify the specific differences in motor control strategies that characterize high-risk crossings compared to low-risk crossings.

The quasi-stiffness of ankle plantarflexion/dorsiflexion (sagittal plane) and eversion/inversion (frontal plane) was calculated by fitting a straight line to the ankle moment vs. the ankle angle curve in the stance phase (Shamaei et al., 2013; Kessler et al., 2020).

EMG data were initially band-pass filtered (cut-off frequency: 10 Hz–500 Hz), then full-wave rectified, and finally low-pass filtered (cut-off frequency: 10 Hz) to generate the linear envelope. The EMG linear envelope was divided by the peak value in all the trials itself for normalization and then interpolated to 101 time-points. The integrated EMG (IEMG) of the TA, SOL, and GL of the stance limb was calculated to reflect the total accumulated muscle activity (Chainok et al., 2022). The values are reported in μV·s.

### 2.4 Statistical analysis

The study design was a 2 × 2 model with two stability levels (good and poor) and two sides (dominant and non-dominant). The Shapiro–Wilk test was used to examine the normality of the data. Levene’s test was used to assess the homogeneity of variances. If the data met the assumption of normality and homogeneity, two-way ANOVA was conducted; otherwise, a non-parametric test was used. Partial correlation analysis was used to examine the relationship between quasi-stiffness and postural stability. A paired sample t-test was used to examine the differences in COM velocity between the DS and NDS obstacle negotiations. The COM velocity in the AP and ML directions was used as a covariate in both the ANOVA and partial correlation analysis. Statistical results included the F-statistic(F), along with partial eta squared (η^2^p) and Cohen’s *d* as the effect sizes. Cohen’s *d* was applied with the following interpretation standards: = 0.8 (large), = 0.5 (medium), and = 0.2 (small) (Sawilowsky, 2009); and η^2^p was applied with conventional thresholds of small (0.01–0.06), medium (0.06–0.14), and large (>0.14) effects (Cohen, 2013). The significance level was set at *p* < 0.05. SPSS 26.0 was used for statistical analysis.

A *post hoc* power analysis was conducted using G*Power to determine the statistical power of the study. Sagittal-plane quasi-stiffness had a significant interaction between postural stability and limb side (η^2^_p_ = 0.133, *p* = 0.025), and the calculated effect size was 0.391. With the sample size 16 and significance level 0.05, the statistical power obtained was 0.829, which exceeds the commonly accepted threshold of 0.80.

## 3 Results

### 3.1 Differences in MoS and ankle quasi-stiffness across conditions

The interaction between postural stability and the side was significant for MoS (F = 2,144.32, *p* < 0.001, and η^2^p = 0.152), frontal-plane quasi-stiffness (F = 7.724, *p* = 0.006, and η^2^p = 0.194), and sagittal-plane quasi-stiffness (F = 5.104, *p* = 0.025, and η^2^p = 0.133) (see Table 2). Both the DS (*p* < 0.001) and NDS (*p* < 0.001) negotiations under the good stability condition exhibited significantly higher MoS than those under the poor stability condition. Additionally, the DS negotiations exhibited significantly higher frontal-plane quasi-stiffness (*p* = 0.001) and sagittal-plane quasi-stiffness (*p* = 0.013) under the poor stability condition than those under the good stability condition, while the NDS negotiations showed no significant difference between the good and poor stability conditions in both frontal-plane quasi-stiffness (*p* = 0.645) and sagittal-plane quasi-stiffness (*p* = 0.515).

**TABLE 2:** Quasi-stiffness and the margin of stability at different conditions.

Variables	DS negotiations		NDS negotiations		Side main effect		Stability main effect		Interaction effect	
	Poor stability	Good stability	Poor stability	Good stability	F	*p*	F	*p*	F	*p*
EV stiffness (Nm/Degree)	1.59 ± 0.55	1.14 ± 0.68	1.11 ± 0.64	1.78 ± 1.04	0.676	0.412	2.135	0.146	5.104	0.025
DF stiffness (Nm/Degree)	3.74 ± 0.84	3.24 ± 0.82	3.15 ± 0.75	3.75 ± 1.31	2.086	0.151	4.265	0.041	7.724	0.006
MoS (cm)	5.98 ± 1.68	10.31 ± 1.61	−3.61 ± 1.30	−1.04 ± 0.08	2,144.32	<0.001	138.312	<0.001	8.205	0.005

Abbreviation: EV, eversion of ankle; DF, dorsiflexion of ankle; MoS, margin of stability; DS, dominant side; NDS, non-dominant side.

### 3.2 Correlation between MoS and quasi-stiffness

**FIGURE 3:**
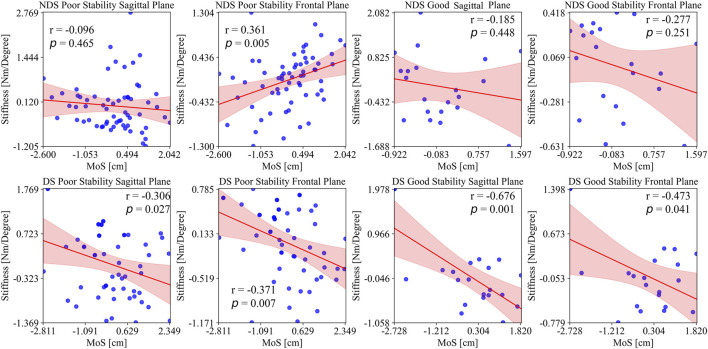
Partial correlation results between quasi-stiffness and margin of stability under different conditions. Blue dots represent data from individual trials. “Good stability” represents trials with the MoS values above the upper quartile, while “poor stability” represents the remaining data. The red solid line represents the linear regression fit; shaded regions indicate the 95% confidence intervals. Abbreviation: NDS, non-dominant side; DS, dominant side; MoS, margin of stability.

During the DS negotiation, a significantly negative correlation was observed between frontal-plane quasi-stiffness (*p* = 0.007 and r = −0.371), sagittal-plane quasi-stiffness (*p* = 0.027 and r = −0.306), and MoS under poor stability conditions and between frontal-plane quasi-stiffness (*p* = 0.041 and r = −0.473), sagittal-plane quasi-stiffness (*p* = 0.001 and r = −0.676), and MoS under good stability conditions (see Figure 3). During the NDS negotiation, a significantly positive correlation was observed between frontal-plane quasi-stiffness (*p* = 0.005 and r = 0.361) and MoS under poor stability conditions (see Figure 3). No significant correlation was observed between quasi-stiffness and MoS under the other conditions.

### 3.3 Differences in muscle activity across the conditions

The interaction between postural stability and the side was significant for IEMG of TA (F = 3.847, *p* = 0.045, and η^2^p = 0.042) (see Table 3). During DS negotiations, muscles exhibited significantly greater IEMG under the poor stability condition than under the good stability condition (*p* = 0.001), while the NDS negotiation showed no significant difference between the good and poor postural stability conditions (*p* = 0.455).

**TABLE 3:** Integrated electromyogram of three muscles at different conditions.

Muscles	DS negotiations		NDS negotiations		Side main effect		Stability main effect		Interaction effect	
	Poor stability	Good stability	Poor stability	Good stability	F	*p*	F	*p*	F	*p*
SOL (μV·s)	197.34 ± 95.29	138.05 ± 41.91	208.63 ± 100.11	154.76 ± 50.34	0.092	0.762	14.339	0.000	0.017	0.897
TA (μV·s)	326.67 ± 68.60	261.38 ± 98.28	310.65 ± 64.68	325.71 ± 63.54	2.152	0.145	7.686	0.006	3.847	0.045
GL (μV·s)	239.53 ± 68.39	245.28 ± 48.33	186.25 ± 86.04	205.86 ± 67.09	11.152	0.001	0.375	0.541	0.291	0.590

Abbreviation: TA, tibialis anterior; SOL, soleus; GL, gastrocnemius lateralis; DS, dominant side; NDS, non-dominant side.

The main effect of postural stability was significant for IEMG of SOL (F = 14.339, *p* < 0.001, and η^2^p = 0.089) (see Table 3). IEMG was significantly greater in the poor stability condition (*p* < 0.001) than in the good stability condition.

The main effect of the side was significant for IEMG of GL (F = 11.152, *p* = 0.001, and η^2^p = 0.071) (see Table 3). The DS negotiations showed significantly greater IEMG than the NDS negotiations (*p* < 0.001).

### 3.4 Differences in COM velocity across the conditions

**FIGURE 4:**
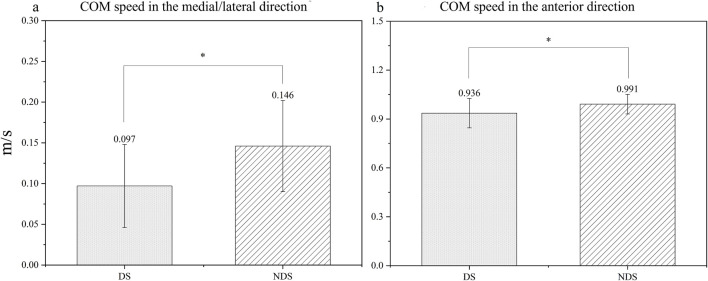
COM velocity under different conditions. Abbreviation: NDS, non-dominant side. DS, dominant side. **(a)** COM speed in the medial/lateral (ML) direction. Note that the absolute values of velocity were used to compare the movement magnitude, accounting for the opposing movement directions between the DS and NDS negotiations. **(b)** COM speed in the anterior/posterior (AP) direction. “*” indicates a statistically significant difference. Error bars represent standard deviations.

The NDS negotiations exhibited significantly faster COM velocity in ML (t = 4.486, *p* < 0.001, and Cohen’s *d* = 0.914) and AP (t = 4.075, *p* < 0.001, and Cohen’s *d* = 0.713) directions than the DS negotiations (see Figure 4).

## 4 Discussion

In this study, when older adults negotiated the obstacle with the DS and NDS, the correlations between ankle quasi-stiffness at the frontal-plane and sagittal-plane and the MoS during DS negotiation were significantly negative, but the correlation between the frontal-plane quasi-stiffness and MoS was significantly positive in NDS negotiation, which contradicts our first hypothesis that assumed the same negative relationship between stiffness and MoS at both DS and NDS conditions. Quasi-stiffness and the activity of muscles functioned differently during DS and NDS negotiations, which is consistent with the second hypothesis. These results are discussed in the following sections.

### 4.1 Relationship between stiffness and the margin of stability during DS negotiation

The frontal-plane and sagittal-plane quasi-stiffness showed a negative correlation with MoS during DS negotiation. Specifically, when older men lift their foot to cross an obstacle, quasi-stiffness increases if the COM moves closer to the COP but decreases if the COM moves away, which is consistent with the results of a previous study on the perturbation test (Smith et al., 2024). We also found that poor stability negotiations had less MoS and greater quasi-stiffness. Ankle quasi-stiffness primarily depends on the viscoelasticity of the muscle–tendon complex and the active properties of muscle contraction (Golkar et al., 2017; Clark and Franz, 2019). Ankle muscle activity increases in narrow-support conditions or when walking on narrow paths with a smaller distance between the COP and COM (Henry et al., 2001; Farrell et al., 2015). The IEMG of the TA, SOL, and GL of the stance limb showed greater activity under poor postural stability conditions. Given that the moments arm of these muscles range from small to moderate in the frontal plane and sagittal plane (McCullough et al., 2011), substantially increased muscle activation is required to effectively enhance ankle stiffness and maintain postural stability. This is supported by previous research indicating that the plantarflexors and dorsiflexors contribute approximately 45% of ankle stiffness during walking (Joshi et al., 2021).

### 4.2 Frontal-plane quasi-stiffness response during NDS negotiation

There was no statistically significant difference in quasi-stiffness between good and poor postural stability during NDS negotiation. However, the correlation analysis revealed an interesting finding: frontal-plane quasi-stiffness positively correlated with the MoS of poor postural stability. Specifically, during NDS negotiation, when the COM moves closer to the COP, quasi-stiffness decreases; when the COM moves away, quasi-stiffness increases. This relationship is contrary to the strategy observed in DS negotiation. This phenomenon is related to the activity of the SOL. Activation of the SOL produces ankle inversion moment and angular velocity during the stance phase, which increases the lateral speed of the COM (Van Le et al., 2021), causing the MoS to remain negative. Meanwhile, the ankle eversion moment and angular velocity decrease, resulting in decreased quasi-stiffness. When the SOL is deactivated, the lateral speed of the COM decreases, MoS becomes positive, and the base of support enlarges. Consequently, the ankle eversion moment and angular velocity increase, leading to increased quasi-stiffness. These factors collectively explain the positive correlation between quasi-stiffness and MoS. There are two potential reasons for SOL activation. First, SOL activity adjusts the position of the COM relative to the COP. Previous research has shown that SOL activation compensates for changes in the distance between the COM and COP caused by foot placement (Van Le et al., 2021). When the MoS is significantly negative, the COM exceeds the body’s support base (Watson et al., 2021), indicating that the stance foot position is closer to the body’s medial side. SOL is activated to shift the COP laterally, thereby enlarging the support area. Second, when the MoS is negative, SOL is activated to generate a greater propulsion force, facilitating faster transition of the body into the next stance phase. This phenomenon occurs exclusively in poor stability trials because the mechanism described above is triggered only when the MoS surpasses a specific negative threshold.

### 4.3 COM velocity reflects differences in the negotiation strategy between DS and NDS

In this study, COM velocity in both the ML and AP directions with the NDS was higher than that with the DS. This difference may be related to the functional roles of the dominant and non-dominant limbs. For right-leg-dominant individuals, the left leg typically serves as the supportive or stabilizing limb, while the right leg assumes the leading or manipulative role (Previc, 1991; Huurnink et al., 2014). During DS negotiation, the stance limb was the left leg, which is more suitable for postural stability but less effective for propulsion (Négyesi et al., 2022). Consequently, older adults require more time to process sensory information and maintain stability while crossing obstacles. They tended to position their body closer to the stance limb (Huang et al., 2008; Wang et al., 2011) and increased ankle quasi-stiffness through muscle contraction to maintain balance. These adjustments help explain the observed correlations between ankle quasi-stiffness and MoS. The longer stance duration also contributed to a decrease in the crossing speed, as shown in our results. In contrast, during NDS negotiation, the stance limb was the right leg, which is more effective for propulsion (Polk et al., 2017). As a result, older adults were able to move their bodies forward more quickly, leading to higher COM velocity. This difference highlights that older adults may adopt different postural strategies depending on the functional role of the stance limb.

## 5 Strength and limitations

The strength of this study is that it is the first to examine the relationship between ankle quasi-stiffness and MoS during DS and NDS obstacle negotiation. However, this study has several limitations. First, only male adults were recruited for the study, which may limit the generalizability of our conclusions. In future studies, female adults will be included to enhance the generalizability. Second, it is important to distinguish between quasi-stiffness and true joint stiffness. Quasi-stiffness, which is defined as the slope of the angle-moment, can be approximated as a linear spring. Therefore, quasi-stiffness should be interpreted as a descriptor of the overall dynamic control of the joint rather than a measure of the intrinsic mechanical stiffness of the actual muscle–tendon units.

## 6 Conclusion

During obstacle negotiation, when using the dominant side, ankle quasi-stiffness was lower to enhance postural stability, whereas when using the non-dominant side, ankle quasi-stiffness was greater to facilitate faster obstacle negotiation. These findings highlight distinct limb-dominance-based strategies when dealing with complex dynamic tasks. Future training methods and assistive device designs should be targeted specifically to the distinct functional roles of each limb.

## Data Availability

The study data are derived from human participants. To ensure compliance with participant consent, privacy protection, and institutional data governance/ethics requirements, we are publicly releasing a de-identified subset that is sufficient for readers to reproduce the full computation workflow and to verify the reported statistical results. Data, results and code used in this article can be downloaded at https://github.com/xuanzewuyan830/obstalces/tree/main and https://zenodo.org/records/18613551.
